# Postoperative management following equine orthopedic surgery: a survey of diplomates of the ACVS and ACVSMR

**DOI:** 10.3389/fvets.2025.1708401

**Published:** 2025-12-05

**Authors:** Carrie Jacobs, Lauren Virginia Schnabel, Caitlyn Redding Horne, Sara Tufts, Emily Gray Medlin Martin, Kim Love

**Affiliations:** 1 Department of Clinical Sciences, North Carolina State University, Raleigh, NC, United States; 2 K. R. Love Quantitative Consulting and Collaboration, Athens, GA, United States

**Keywords:** postoperative, equine, rehabilitation, orthopedic, surgery

## Abstract

Postoperative management, including rehabilitation and physical therapy, is important to decrease pain and improve return to function in human and small animal orthopedic surgical cases; however, recommendations for postoperative management for equine orthopedic surgical cases is limited. As the field of equine rehabilitation continues to expand, we must understand how postoperative management and rehabilitation modalities are being used to determine evidence based guidelines for commonly utilized modalities. The objectives of this cross-sectional survey were to (1) investigate postoperative management recommendations for four common equine orthopedic surgical scenarios by diplomates of the American College of Veterinary Surgeons (ACVS) and American College of Veterinary Sports Medicine and Rehabilitation (ACVSMR) and to (2) determine if recommendations were different between specialties and (3) different between surgical scenarios. An electronic cross-sectional survey with four equine orthopedic surgical scenarios (simple arthroscopy [SA], septic arthritis [SJ], deep digital flexor tendon tear [DT], and neurectomy of the deep branch of the lateral plantar nerve and fasciotomy [NF]) with questions regarding postoperative management recommendations was distributed to diplomates of the ACVS and ACVSMR. A total of 85 surveys were completed. Non-steroidal anti-inflammatory administration, bandaging, hand-walking, and small paddock turnout, were most recommended for all scenarios. SA, SJ, and NF cases had small paddock turnout, full turnout, and ridden exercise recommended sooner than DT cases. Longer periods of hand-walking and small paddock turnout were recommended for DT cases. Intrathecal therapies were most frequently recommended for DT cases. ACVSMR diplomates were more likely to recommend rehabilitation modalities for certain scenarios. In conclusion, results of this survey describe postoperative management for equine orthopedic surgical cases recommended by ACVS and ACVSMR diplomates. Few differences were identified in recommendations between diplomates. Differences were identified between the different surgical case scenarios.

## Introduction

Postoperative management, including rehabilitation and physical therapy, contribute to decreased pain and improved return to function in human and small animal orthopedic surgical cases (1–4). Human orthopedic surgery patients undergoing enhanced recovery after surgery (ERAS) protocols, which include passive and active rehabilitation exercises, gradual increases in standing and walking, cryotherapy, analgesic medications, and physical therapy, had shorter hospital stays, improved joint function recovery scores, lower incidence of complications, and higher patient satisfaction (2, 3). Dogs undergoing tibial plateau leveling osteotomy (TPLO) surgery combined with rehabilitation plans including passive range of motion exercise, gradually increased leash walking, cryotherapy, and a diet with omega-3 fatty acids, had improved peak vertical forces, lower lameness scores, and decreased progression of osteoarthritis compared to dogs that did not undergo a rehabilitation plan (4, 5).

Despite research on rehabilitation modalities in humans, small animals, and horses, there is minimal literature specifically regarding postoperative management and the use of rehabilitation modalities for horses. In horses undergoing orthopedic surgery, rest and non-steroidal anti-inflammatory drugs (NSAIDs) are mainstays of postoperative management, however information such as clinician choice of medication, dosing, length and frequency of administration is lacking (6). In one of the few studies evaluating rehabilitation modalities following elective arthroscopic surgery, Thoroughbred racehorses undergoing underwater treadmill rehabilitation were more likely to return to racing and had shorter lay-up times compared to horses undergoing a conventional postoperative rehabilitation plan (7). Another study evaluating core abdominal rehabilitation exercises in postoperative colic patients found cases undergoing core exercises returned to work and training faster compared to control horses (8).

As the field of equine rehabilitation continues to expand, there is a need to determine evidence based guidelines for commonly utilized postoperative management and rehabilitation modalities. Similar to human and small animal orthopedic surgery patients, equine patients are likely to benefit from rehabilitation in the postoperative period not only to decrease pain and lameness secondary to their procedures but also to maintain fitness to promote an earlier return to function.

The objectives of this cross-sectional survey were to (1) investigate postoperative management recommendations for four common equine orthopedic surgical scenarios by diplomates of the American College of Veterinary Surgeons (ACVS) and American College of Veterinary Sports Medicine and Rehabilitation (ACVSMR) and to (2) determine if recommendations were different between specialties and (3) different between surgical scenarios.

## Materials and methods

The survey and recruitment materials were approved by the North Carolina State University Institutional Review Board (IRB 25925). The surgical scenarios and survey questions were developed by 4 co-authors (CJ, LS, CH, ST) and evaluated by a separate co-author (EM) to determine time to survey completion and question modification.

Contact regarding survey participation was made exclusively through email. The recruitment email indicated the purpose of the study and that participation was voluntary. Additional information included approximate length of time to complete the survey, that participation and responses were anonymous, and contact information for the primary investigator (CJ) and the North Carolina State University Institutional Review Board. The recruitment email and survey were distributed using Qualtrics (qualtrics.com; Seattle, WA) on September 19, 2023, and included an individual link to access informed consent and the survey. Two additional emails were sent at 2 and 4 weeks following the initial email, reminding diplomates of the survey. The survey was closed on November 1, 2023.

The invited survey population included large animal diplomates of ACVS or ACVSMR. A list of ACVS diplomates was created by searching for ‘large animal’ and ‘equine’ diplomates with an active email address using the ACVS Diplomate Search function on the ACVS website. A list of ACVSMR diplomates was created by searching for ‘equine’ diplomates with an active email address using the ACVSMR Diplomate Search function on the ACVMSR website. Diplomates that were double boarded (ACVS, ACVSMR) were included only once, on the ACVS email list.

The survey included questions related to respondent demographics including years in practice, type of practice, board certification (ACVS, ACVSMR, or both), and the discipline of horse most often treated. Following this, four surgical scenarios were presented (Supplementary material 1), with the same set of 32 questions following each surgical scenario (Supplementary material 2). The scenarios included brief descriptions and select imaging or pictures of the cases. The scenarios included arthroscopic removal of a simple osteochondral fragment, arthroscopy for treatment of a septic joint secondary to a puncture wound, tenoscopy of the digital flexor tendon sheath for a tear of the deep digital flexor tendon, and neurectomy of the deep branch of the lateral plantar nerve and fasciotomy for bilateral hindlimb proximal suspensory desmitis. Questions following the scenarios included those regarding type and length of administration of NSAIDs; the use and length of time for strict stall rest, hand-walking, small paddock and full turnout exercise, and ridden exercise; the use of intra-articular/intra-thecal/intra-lesional therapies; the use and length of time for bandaging; and the use, type, and length of time for multiple rehabilitation modalities (cryotherapy, core strengthening exercise, laser therapy, pulsed electromagnetic field therapy [PEMF], extracorporeal shockwave therapy [ESWT], and range of motion exercise). All questions were presented in the same order after each scenario, except for certain questions that were only displayed if the previous question had been answered in a specific way. For example, if the question ‘Do you recommend hand-walking?’ was answered yes, then additional questions regarding hand-walking would be displayed. If this was answered no, then the respondent would continue through the survey. Questions regarding choice of NSAID, choice of intra-articular/intra-thecal/intra-lesional therapies, type of cryotherapy, and type of PEMF allowed respondents to select ‘other’ and provide an alternate answer.

Response rate was considered as the number that started the survey divided by the number contacted. The completion rate was considered the number of completed surveys divided by the number that started the survey.

Summary statistics were prepared using IBM SPSS Statistics v. 29. All statistical analyses were performed using R software (Version 4.2.3). Statistical significance was set at *p* < 0.05. To compare binary (yes/no) responses between scenarios, mixed effects binary logistic regression models were used. These included a random intercept of respondents to account for the repeated responses from the same individuals in different scenarios. When significant differences were found, Tukey-adjusted *post hoc* pairwise comparisons were performed. Ordinal scenario responses (ie. total time recommended for treatment, time to initiate treatment) were analyzed using mixed effects ordinal logistic regression models, with Tukey-adjusted pairwise comparisons were conducted following significant findings.

Comparisons of binary responses between diplomate groups were analyzed using Fisher’s exact tests. When these were significant, *post hoc* pairwise Fisher’s exact tests using a Holm adjustment were applied. Ordinal variables between diplomate groups were assessed using Kruskal-Wallis tests, with *post hoc* Dunn’s pairwise comparisons tests using a Holm adjustment performed when significant differences were identified.

## Results

Seven hundred and fifty-four emails were sent (644 to ACVS diplomates, 110 to ACVSMR diplomates, and 60 to diplomates of both ACVS and ACVSMR) with 44 emails ‘returned to sender’ for a total of 710 diplomates that received the recruitment email and survey link. The response rate was 15.1% (107/710) and of those who participated in the study, the completion rate was 79.4% (85/107). Only results from the 85 completed surveys are reported. Results of demographic questions are displayed in Table 1.

**Table 1:** Survey respondent demographics.

	ACVS	ACVSMR	ACVS+ACVSMR
Diplomate type	64/85	8/85	13/85

	<5 years	5–10 years	11–20 years	>20 years
Years in practice	1/85	22/85	34/85	28/85

	Private			Academic		Industry
	Equine only	Mixed	Large animal	Equine only	Large animal	
Practice type	43/85	1/85	1/85	19/85	20/85	1/85

	Sport horses	Western performance	Show horses	Racehorse	Pleasure	Combination of disciplines
Equine discipline	18/85	6/85	0/85	10/85	0/85	51/85

### Non-steroidal anti-inflammatory drug administration

A majority (85.9–92.9%) recommended NSAIDs to 100% of horses for all scenarios (simple arthroscopy [SA], septic joint [SJ], deep digital flexor tendon tear [DT], and neurectomy of the deep branch of the lateral plantar nerve and fasciotomy [NF]). Phenylbutazone was most recommended (78.6–91.8%) and was recommended more for SA compared to SJ (*p* = 0.002), DT (*p* = 0.001), and NF (*p* = 0.002). A shorter course of NSAIDs (0–3 or 4–7 days) was recommended for SA (95.3%) compared to SJ (65.9%), DT (76.2%), and NF (84.7%). A shorter course of NSAIDs (0–3 days, 4–7 days) was also recommended for NF compared to SJ cases (*p* = 0.006) (Table 2).

**Table 2:** Recommendations for NSAID use, type of NSAID, and length of NSAID administration per case scenario.

% cases NSAIDs recommended	100%	99–75%	74–50%	49–25%	24–0%	*p*-value
						0.207
Simple arthroscopy	73	3	5	1	3	
Septic joint	77	4	2	0	2	
Deep digital flexor tendon tear	78	1	0	2	3	
DBLP neurectomy/fasciotomy	76	3	4	2	0	

Type of NSAID recommended	PB*	FM*	FC*	Similar frequency PB and FM*	Similar frequency PB, FM, FC*	Other	*p*-value
							0.003
Simple arthroscopy	78	5	2	0	0	0	referent
Septic joint	67	6	2	8	0	1	0.002
Deep digital flexor tendon tear	66	7	3	5	1	2	0.001
DBLP neurectomy/fasciotomy	68	7	1	5	3	1	0.002

Time of NSAID administration	0-3d	4-7d	8-12d	13-14d	15-21d	> 21d	*p*-value	*p*-value
							<0.001	
Simple arthroscopy	31	50	4	0	0	0	referent	
Septic joint	6	50	21	5	3	0	<0.001	0.006
Deep digital flexor tendon tear	13	51	15	1	0	4	<0.001	
DBLP neurectomy/fasciotomy	11	61	9	2	0	2	<0.001	referent

*PB, phenylbutazone; FM, flunixin meglumine; FC, firocoxib.

More ACVS diplomates (89.1%) recommended NSAIDs to all patients undergoing SA compared to ACVSMR diplomates (55.6%, *p* = 0.032), though no difference in NSAID type was present between specialties for any scenario. There was no difference between the length of NSAID administration recommended between specialties for SA, SJ, or NF. ACVS diplomates recommend NSAIDs for longer periods for DT compared to ACVS/ACVSMR diplomates (*p* = 0.011).

### Stall rest

Cases undergoing SA were more likely to have shorter periods of stall rest compared to SJ (*p* = 0.047), DT (*p* = 0.002), and NF (*p* < 0.001). Stall rest was recommended for 0–2 weeks by 70.6% of respondents for SA compared to 48.2–58.3% of respondents for SJ, DT, and NF. No differences were present between specialties for total recommended time for stall rest for any scenario (Table 3).

**Table 3:** Time to initiate different types of exercise by case scenario.

	0-2w	3-4w	5-6w	7-8w	9-10w	11-12w	13-15w	>16w	*p*-value	*p*-value
Time to initiate hand walking									0.003	
Simple arthroscopy	34	42	1	0	0	0	0	0	0.011	
Septic joint	40	36	4	0	0	0	0	0	0.023	
Deep digital flexor tendon tear	57	20	5	0	0	0	0	1	referent	
DBLP neurectomy/fasciotomy	40	36	4	1	1	0	0	0	0.012	
Time to initiate small paddock exercise									<0.001	
Simple arthroscopy	2	28	31	14	3	0	0	0	<0.001	<0.001
Septic joint	0	34	25	13	4	1	0	0	<0.001	<0.001
Deep digital flexor tendon tear	1	5	15	23	10	7	2	9	referent	
DBLP neurectomy/fasciotomy	2	13	21	16	8	3	3	4	0.004	referent
Time to initiate full turnout exercise									<0.001	
Simple arthroscopy	0	5	19	31	17	10	2	1	<0.001	<0.001
Septic joint	0	3	17	32	12	18	2	1	<0.001	<0.001
Deep digital flexor tendon tear	0	1	3	6	11	19	5	39	referent	
DBLP neurectomy/fasciotomy	0	3	4	16	18	15	7	22	0.001	referent
Time to initiate ridden exercise									<0.001	
Simple arthroscopy	0	1	19	25	19	14	6	1	<0.001	<0.001
Septic joint	0	1	12	23	22	16	5	6	<0.001	0.013
Deep digital flexor tendon tear	0	3	2	8	8	16	13	33	referent	
DBLP neurectomy/fasciotomy	0	7	3	18	13	13	7	24	0.005	referent

### Hand-walking exercise

Most respondents (90.6–98.8%) recommended hand-walking exercise for all scenarios. More recommended hand-walking for DT (98.8%) compared to SA (90.6%) (*p* = 0.033). Respondents recommended hand-walking be initiated sooner for DT compared to SA (*p* = 0.011), SJ (*p* = 0.023), and NF (*p* = 0.012) (Table 4). Longer periods of hand-walking were recommended for DT compared to SA (*p* < 0.001), SJ (*p* < 0.001), and NF (*p* = 0.015). Additionally, NF had longer periods of hand-walking recommended compared to SA (*p* < 0.001) and SJ (*p* < 0.001) (Table 3).

**Table 4:** Total amount of time different types of exercise recommended by case scenario.

	0-2w	3-4w	5-6w	7-8w	9-10w	11-12w	13-15w	>16w	*p*-value	*p*-value
Total time stall rest									<0.001	
Simple arthroscopy	60	24	1	0	0	0	0	0	referent	
Septic joint	47	32	3	3	0	0	0	0	0.047	
Deep digital flexor tendon tear	49	19	3	6	1	2	1	4	0.002	
DBLP neurectomy/fasciotomy	41	27	7	7	0	2	0	1	<0.001	
Total time hand walking									<0.001	
Simple arthroscopy	16	40	9	11	0	0	0	1	<0.001	<0.001
Septic joint	12	44	11	8	2	2	0	1	<0.001	<0.001
Deep digital flexor tendon tear	2	16	16	32	1	8	2	6	referent	
DBLP neurectomy/fasciotomy	6	26	15	21	2	4	1	7	0.015	referent
Total time small paddock exercise									<0.001	
Simple arthroscopy	16	46	5	9	0	0	1	0	<0.001	<0.001
Septic joint	9	50	6	9	1	3	0	0	<0.001	<0.001
Deep digital flexor tendon tear	1	26	13	17	3	7	2	3	referent	
DBLP neurectomy/fasciotomy	4	27	12	17	4	6	1	0		referent

ACVSMR diplomates recommended hand-walking longer than ACVS diplomates for SA (*p* = 0.011). There were no differences between specialties for the other scenarios.

### Small paddock exercise

Most respondents (83.5–91.8%) recommended small paddock exercise for all scenarios.

Respondents recommended small paddock exercise be initiated sooner for SA (*p* < 0.001), SJ (*p* < 0.001), and NF (*p* = 0.004) compared to DT (Table 4). Small paddock exercise was initiated sooner for SA (*p* < 0.001) and SJ (*p* < 0.001) compared to NF. Small paddock exercise was recommended for longer periods for NF and DT compared to SA (both *p* < 0.001) and SJ (both *p* < 0.001) (Table 4).

More ACVS diplomates recommended small paddock exercise for SJ compared to ACVSMR diplomates (*p* = 0.002). There were no differences between specialties for the other scenarios.

### Full turnout exercise

Respondents recommended full turnout be initiated sooner for SA (*p* < 0.001), SJ (*p* < 0.001), and NF (*p* = 0.004) compared to DT (Table 4). Additionally, SA (*p* < 0.001) and SJ (*p* < 0.001) had turnout recommended sooner compared to NF. For SA and SJ, a majority (57.6–58.9%) recommended full turnout at 5–6 or 7–8 weeks. For NF, a majority (57.6%) recommended full turnout from 7 to 12 weeks. For DT, 52.4% recommended full turnout from 13 to 15 weeks or >16 weeks.

No differences were present between specialties for time to initiate full turnout exercise for any scenario.

### Ridden exercise

Return to ridden exercise was recommended sooner for SA (*p* < 0.001), SJ (*p* < 0.001), and NF (*p* = 0.005) compared to DT (Table 4). Additionally, ridden exercise was recommended sooner for SA (*p* < 0.001) and SJ (*p* < 0.001) compared to NF cases. No differences were present between specialties for time to initiate ridden exercise for any scenario.

### Intra-articular, intrathecal, and intra-lesional therapies

Intrathecal therapies were recommended more for DT compared to intra-articular therapies for SA (*p* = 0.001) and SJ (*p* = 0.001) and intra-lesional therapies for NF (*p* < 0.001). There was no difference in the recommendation (yes/no) for using these therapies between SA, SJ, and NF. When these therapies were recommended, SJ had longer times to initiate these treatments following surgery compared to DT (*p* = 0.003) and NF (*p* = 0.001). Hyaluronate sodium was recommended most for SA (38.2%) and DT (23.7%), autologous protein solution (APS, 21.4%) for SJ, and platelet rich plasma (PRP, 63.3%) for NF. There were no differences between specialties in recommending intra-articular, intrathecal, or intra-lesional for any surgical scenario.

### Bandaging

Most respondents recommended bandaging for all scenarios (95.3–98.9%) (Table 5). Bandaging was recommended for a shorter period for SA compared to SJ (*p* = 0.005), DT (*p* < 0.001), and NF (*p* = 0.001) (Table 6). Bandaging was also shorter for SJ compared to DT (*p* = 0.014). ACVSMR diplomates recommended longer periods of bandaging for DT compared to ACVS diplomates (*p* = 0.034). There were no differences between specialties for the other scenarios (Table 7).

**Table 5:** Recommendations for rehabilitation modalities per scenario.

	Yes	No	*p*-value	*p*-value
Bandaging			0.292	
Simple arthroscopy	82	3		
Septic joint	83	2		
Deep digital flexor tendon tear	83	1		
DBLP neurectomy/fasciotomy	81	4		
Cryotherapy			0.001	
Simple arthroscopy	9	76	referent	
Septic joint	21	64	0.009	
Deep digital flexor tendon tear	28	55	<0.001	
DBLP neurectomy/fasciotomy	24	60	<0.001	
Core strengthening exercises			0.005	
Simple arthroscopy	21	64	0.003	0.009
Septic joint	23	60	0.011	0.011
Deep digital flexor tendon tear	31	53		referent
DBLP neurectomy/fasciotomy	33	52	referent	
Laser therapy			<0.001	
Simple arthroscopy	6	79	referent	
Septic joint	15	70	0.025	referent
Deep digital flexor tendon tear	28	56	<0.001	0.004
DBLP neurectomy/fasciotomy	23	62	<0.001	
Pulsed electromagnetic field therapy			0.512	
Simple arthroscopy	3	82		
Septic joint	3	82		
Deep digital flexor tendon tear	5	78		
DBLP neurectomy/fasciotomy	5	80		
Extracorporeal shockwave therapy			<0.001	
Simple arthroscopy	0	85	<0.001	0.003
Septic joint	2	83	<0.001	0.001
Deep digital flexor tendon tear	22	61	<0.001	referent
DBLP neurectomy/fasciotomy	52	33	referent	
Range of motion exercise			<0.001	
Simple arthroscopy	40	45	<0.001	0.028
Septic joint	53	32	0.010	Referent
Deep digital flexor tendon tear	66	17	referent	
DBLP neurectomy/fasciotomy	48	37	0.001	

**Table 6:** Recommendation for rehabilitation modalities where significant differences identified between specialties.

	Yes	No	*p*-value	*p*-value
Cryotherapy – simple arthroscopy			<0.001	
ACVS	2	62	<0.001	
ACVSMR	5	4	referent	
ACVS/ACVSMR	2	10		
Cryotherapy – septic joint			<0.001	
ACVS	9	55	<0.001	
ACVSMR	7	2	referent	
ACVS/ACVSMR	5	7		
Cryotherapy – DDFT tear			<0.001	
ACVS	14	48	0.006	
ACVSMR	7	2	referent	
ACVS/ACVSMR	7	5		
Cryotherapy – DBLPN			<0.001	
ACVS	11	52	0.013	0.013
ACVSMR	6	3	referent	
ACVS/ACVSMR	7	5		referent
Core strengthening exercises – simple arthroscopy			0.009	
ACVS	12	52	0.017	
ACVSMR	6	3	referent	
ACVS/ACVSMR	3	9		
Core strengthening exercises – septic joint			0.005	
ACVS	12	50	0.019	
ACVSMR	6	3	referent	
ACVS/ACVSMR	5	7		
Core strengthening exercises – DDFT tear			0.004	
ACVS	17	46	0.051	0.048
ACVSMR	6	3	referent	
ACVS/ACVSMR	8	4		referent
Laser - DDFT			<0.001	
ACVS	14	49	0.006	
ACVSMR	7	2	referent	
ACVS/ACVSMR	7	5		
PEMF – septic joint			0.007	
ACVS	0	64	0.041	
ACVSMR	2	7	referent	
ACVS/ACVSMR	1	11		
Range of motion – simple arthroscopy			0.027	
ACVS	27	37	0.035	
ACVSMR	8	1	referent	
ACVS/ACVSMR	5	7		

**Table 7:** Total time recommended for rehabilitation modalities.

	0-2w	3-4w	5-6w	7-8w	9-10w	11-12w	13-15w	>16w	*p*-value	*p*-value
Bandaging									<0.001	
Simple arthroscopy	65	15	1	1	0	0	0	0	referent	
Septic joint	48	31	0	3	0	0	0	0	0.005	referent
Deep digital flexor tendon tear	37	32	7	6	1	0	0	0	<0.001	0.014
DBLP neurectomy/fasciotomy	47	26	4	4	0	0	0	0	0.001	
Cryotherapy									0.011^a^	
Simple arthroscopy	5	4	0	0	0	0	0	0		
Septic joint	9	11	0	0	0	0	0	1		
Deep digital flexor tendon tear	9	13	2	1	2	1	0	0		
DBLP neurectomy/fasciotomy	6	13	1	3	0	1	0	0		
Core strengthening									0.003	
Simple arthroscopy	0	4	5	4	0	2	0	6		
Septic joint	0	6	6	4	1	1	0	5	referent	
Deep digital flexor tendon tear	0	5	6	5	0	5	0	10	0.006	
DBLP neurectomy/fasciotomy	0	4	8	6	1	4	1	9	0.008	
Laser therapy									0.150	
Simple arthroscopy	2	4	0	0	0	0	0	0		
Septic joint	4	9	1	0	1	0	0	0		
Deep digital flexor tendon tear	5	11	5	5	1	0	0	0		
DBLP neurectomy/fasciotomy	3	12	5	0	1	2	0	0		
Extracorporeal shockwave^b^									0.273	
Simple arthroscopy	0	0	0	0	0	0	0	0		
Septic joint	1	0	1	0	0	0	0	0		
Deep digital flexor tendon tear	1	7	8	2	1	2	0	0		
DBLP neurectomy/fasciotomy	1	21	20	6	2	2	0	0	referent	
Range of motion									<0.001	
Simple arthroscopy	6	17	6	6	1	0	0	4	<0.001	<0.001
Septic joint	5	20	10	11	2	3	0	2	<0.001	<0.001
Deep digital flexor tendon tear	6	13	16	11	3	8	1	8		referent
DBLP neurectomy/fasciotomy	1	11	8	5	2	12	0	9	referent	

a,b, not significant.

### Cryotherapy

Cryotherapy was not commonly recommended for any scenario with recommendations lower for SA (10.6%) compared to SJ (24.7%, *p* = 0.009), DT (33.7%, *p* < 0.001), and NF (28.6%, *p* < 0.001) (Table 5). When cryotherapy was recommended, a commercial dry sleeve/compression device (44.4–50.0%) was the preferred method. In most scenarios, cryotherapy was recommended during 0–2 and 3–4 weeks, and while the *p*-value for the logistic regression indicated significant differences (*p* = 0.011) in total time cryotherapy is recommended, when Tukey-adjusted *post hoc* pairwise comparisons were performed, no differences were identified (Table 6). ACVSMR diplomates were more likely to recommend cryotherapy for SA (*p* < 0.001), SJ (*p* < 0.001), DT (*p* = 0.006), and NF (*p* = 0.013) compared to ACVS diplomates. Diplomates of both specialties were more likely to recommend cryotherapy for NF (*p* = 0.013) compared to ACVS-only diplomates (Table 7).

### Core strengthening exercises

Most respondents (61.2–75.3%) did not recommend core strengthening exercises for any scenario (Table 5) and were recommended more for DT and NF compared to SA (*p* = 0.009, *p* = 0.003) and SJ (*p* = 0.011, *p* = 0.011). When core strengthening exercises were recommended, DT (*p* = 0.006) and NF (*p* = 0.008) were recommended longer periods compared to SA = (Table 6). ACVSMR diplomates were more likely to recommend core strengthening exercises for SA (*p* = 0.017) and SJ (*p* = 0.019) compared to ACVS diplomates (Table 7). For DT, the recommendation was different between diplomates of both specialties and ACVS-only diplomates (*p* = 0.048). For NF, there was no difference among diplomates in the recommendation of core strengthening exercises.

### Laser therapy

Laser therapy was not commonly recommended (7.1–33.3%) (Table 5). Recommendations for laser therapy was lower for SA compared to SJ (*p* = 0.025), DT (*p* < 0.001), and NF (*p* < 0.001) and for SJ compared to DT (*p* = 0.004). When recommended, high level (class 4) laser was more likely to be recommended for DT and NF cases when compared to SJ (*p* = 0.004, *p* < 0.001). No differences were identified in the amount of time laser therapy was recommended (Table 6). ACVSMR diplomates were more likely to recommend laser therapy compared to ACVS diplomates (*p* = 0.006) for DT (Table 7). There were no differences between specialties for the other surgical scenarios.

### Pulsed electromagnetic field therapy (PEMF)

The recommendation for PEMF (3.5–6%) was low and when recommended, the preferred method was using a PEMF wand (60–80%) (Table 5). No difference was found between scenarios for the total time PEMF therapy was recommended; however, this was likely due to the small percentage who recommended PEMF. ACVSMR diplomates were more likely to recommend PEMF for SJ compared to ACVS diplomates (*p* = 0.041). There were no differences between specialties for the other scenarios (Table 7).

### Extracorporeal shockwave therapy (ESWT)

Shockwave therapy was recommended more for NF compared to SA (*p* < 0.001), SJ (*p* < 0.001), and DT (*p* < 0.001) (Table 5). It was recommended more in DT compared to SA (*p* = 0.003) and SJ (*p* = 0.001). The total time of shockwave therapy was not different between NF and DT (*p* = 0.273) (Table 6). Most recommended the total time of shockwave therapy to be over 3–4 and 5–6 weeks (71.4% DT, 78.9% NF). No differences were present between specialties for the recommendation of or total time to recommend shockwave therapy between scenarios (Table 7).

### Range of motion exercises (ROM)

Recommendation for ROM exercises was variable (47.1–79.5%) (Table 5) across scenarios but was greater for DT cases was greater compared to SA (*p* < 0.001), SJ (*p* = 0.010), and NF (*p* < 0.001). Recommendation of ROM was also greater for SJ compared to SA (*p* = 0.028). Time to perform ROM was higher for DT and NF compared to SA (*p* < 0.001, *p* < 0.001) and SJ (*p* < 0.001, *p* < 0.001) (Table 6). ACVSMR diplomates recommended ROM for SA more compared to ACVS diplomates (*p* = 0.035). There were no differences between specialties for the recommendation of ROM exercises for other scenarios (Table 7). ACVSMR diplomates and diplomates of both specialties recommended ROM exercises for longer periods of time for SJ compared to ACVS-only diplomates (*p* = 0.032, *p* = 0.047).

## Discussion

This study presents survey results for postoperative management recommendations for four commonly performed equine orthopedic surgeries among ACVS and ACVSMR diplomates. Rehabilitation modalities to treat equine athletes surveyed among a wide variety of equine veterinary groups has previously been reported; however, this is the first looking specifically at recommendations by ACVS and ACVSMR diplomates following postoperative equine orthopedic surgery (9).

For all scenarios, NSAID administration (85.9–92.9%), bandaging (95.3–98.8%), hand-walking exercise (90.6–98.8%), and small paddock turnout (83.5–91.8%) were recommended by most survey respondents. Similarly, Wilson et al. found controlled hand-walking (97.3%) and compression bandaging (89.5%) to be the most used rehabilitation modalities, though this study did not evaluate the use of NSAIDs or small paddock turnout (9). Phenylbutazone was the most recommended NSAID among respondents for all scenarios, which is consistent with previous reports that phenylbutazone is the most prescribed NSAID for orthopedic pain in equine practice (10). It is important to note, that studies evaluating the differences between phenylbutazone and flunixin meglumine, a similar non-selective COX inhibitor, have found few differences between the two drugs for treating lameness (11–13). Similarly, a study evaluating phenylbutazone and firocoxib, a selective COX-2 inhibitor, found no difference in efficacy for reducing lameness in horses secondary to osteoarthritis (14).

Shorter periods of bandaging were recommended for SA compared to SJ, NF, and DT. Additionally, most forms of exercise including small paddock, full turnout, and ridden exercise, were recommended to be initiated for SA, SJ, and NF cases sooner than DT cases. Hand-walking exercise was the exception and was recommended to be started sooner in the postoperative period for DT compared to SA, SJ, and NF.

The differences in bandaging and exercise recommendations between scenarios are likely secondary to differences in tissue type affected by the surgical procedures, innate differences in healing time and healing capabilities for these tissues, and differing goals of the surgical procedures. For instance, articular cartilage, subchondral bone, and synovium of joints are affected in SA and SJ whereas tendon and synovium of the digital flexor tendon sheath are affected in DT cases, and the suspensory ligament and deep branch of the lateral plantar nerve are affected in NF. And while decreasing inflammation and returning affected joints to a homeostatic state is a goal in SA and SJ cases, the inflammatory insult and intensity of inflammation are likely to differ between cases undergoing elective arthroscopy versus arthroscopy for a septic joint (15). Similarly, while DT and NF cases both involve soft tissue structures, goals of surgery in DT include removal of torn tendon fibers to decrease inflammation, promotion of tendon healing, and reduction of adhesion formation, while goals of NF surgery are to decrease pain secondary to ligament enlargement and compression (16, 17). Earlier initiation of hand-walking exercise in DT compared to the other surgical procedures is likely to promote tendon movement within the sheath to decrease adhesion formation between the sheath and the injured tendon while also using mobilization and a gradual increase in stress to improve the quality of the healing tissue (18).

Intra-thecal therapies were recommended more for DT compared to a similar treatment (intra-articular or intra-lesional therapy) in SA, SJ, and NF. Recommendations were variable across surgical scenarios, however hyaluronate sodium was the most recommended intra-articular or intra-thecal therapy in SA and DT. An older study evaluating tendon healing and adhesion development in a collagenase-induced tendonitis model within the digital flexor tendon sheath found improved ultrasonographic evidence of tendon healing following hyaluronate sodium injection into the digital flexor tendon sheath (19). Additionally, horses who had the digital flexor tendon sheath medicated with hyaluronate sodium following induction of tendon lesions had fewer and smaller adhesions on gross pathologic evaluation compared to horses with sheaths medicated with methylcellulose gel. Results of this study may be one of the reasons why hyaluronate sodium was the most recommended intrathecal therapy in the DT case scenario.

Autologous protein solution (APS) was the commonly recommended intra-articular therapy in SJ. Similarly, studies of equine practitioners and diplomates found intra-articular administration of APS to be commonly recommended in cases with acute and chronic articular pathology and in cases with cartilage injury (20, 21). This is not surprising as APS has been shown to decrease gross and histologic effects of acute inflammation on cartilage and the synovial membrane in an experimental model (22). Intra-lesional platelet rich plasma (PRP) was most recommended for NF, which is consistent with other surveys that show PRP is commonly used for ligament and tendon injuries (10, 20, 21). Additionally, recent literature reports improved outcomes for horses with proximal suspensory disease treated with PRP (23, 24).

Between scenarios, recommendations varied for the use of cryotherapy, core strengthening exercises, laser therapy, PEMF, ESWT, and ROM exercises and were recommended more in DT or NF scenarios. The exception was ROM exercises, which were more likely to be recommended for DT and SJ. Wilson et al. found higher utilization of laser therapy, shockwave therapy, and pulsed electromagnetic field therapy (PEMF) for tendon and ligament injury and in post arthroscopy cases compared to our results (9).

Despite evidence for cryotherapy use in prevention and management of laminitis, minimal literature exists on the effect of cryotherapy and its utility for postoperative equine orthopedic cases (25, 26) and may explain the lower and variable recommendation of cryotherapy for the case scenarios. Following canine stifle surgery, cryotherapy decreased swelling and improved weight bearing, pain scores, and stifle joint range of motion (5, 27–29). Cryotherapy may improve pain and recovery in equine patients in the acute postoperative period, though additional research is warranted.

Less than half of respondents recommended core strengthening exercises for any of the common orthopedic surgical scenarios. This is surprising as equine patients undergoing core abdominal rehabilitation exercises for 4 weeks following abdominal exploratory surgery returned to work faster when compared to controls (9). While results of this study involve horses with abdominal incisions and prolonged rest, these exercises may be beneficial to horses following orthopedic surgery, particularly those undergoing prolonged periods of rest, to prevent back pain and promote return to function sooner.

Recent studies have found favorable results for the use of high-power laser therapy (Class IV laser) for equine soft tissue injuries such as superficial digital flexor tendinopathy, deep digital flexor tendinopathy, suspensory ligament body and suspensory ligament branch injuries (30–33). Given that most laser research in horses has focused on soft tissue injuries, it is not surprising that our survey results found laser therapy to be most recommended for DT and NF.

Few studies have evaluated the effect of PEMF therapy in equine musculoskeletal injury and minimal effects have been found on back pain, bone isotope uptake, and incorporation of cancellous bone grafts (34–36). Given this, it is not surprising recommendations for PEMF therapy were low among survey respondents.

The greater recommendation for ESWT in NF and DT cases compared to SA and SJ is not surprising as its use for equine soft tissue injuries, particularly those of the suspensory ligament, is supported in the literature (23, 37–39). Dogs treated with ESWT following TPLO had improved weight bearing with no detrimental effects to osteotomy healing (40). ESWT may improve pain and recovery of horses following orthopedic surgery, however additional research is needed.

Recommendation for ROM was greater for DT cases compared to other surgical scenarios despite studies to support the use of ROM exercises to improve lameness, synovial membrane inflammation, and likelihood to return to racing in horses with joint pathology and following arthroscopy (7, 41). There are multiple methods to accomplish ROM and their effects on joint kinematics have been studied (42–44); however we did not specify type of ROM exercise in our survey and this may have affected responses.

Between ACVS and ACVSMR diplomates, there were few differences between recommendations. For example, regarding hand-walking exercise, ACVSMR diplomates recommended longer periods of hand-walking exercise for SA compared to ACVS diplomates. ACVSMR diplomates were more likely to recommend rehabilitation modalities such as cryotherapy, core strengthening exercises, laser therapy, PEMF, and ROM, compared to ACVS diplomates for certain scenarios. The variation seen in recommendations across scenarios, between diplomates within this study and between the multiple different studies cited throughout this manuscript, are likely due to a variety of factors including the types of cases presented, the healing capacities of the affected tissues, the quality and availability of supporting literature, variability in clinician training and experience with rehabilitation modalities and therapies, variability in accessibility of rehabilitation modalities, and associated cost.

There are several limitations of this study. First, the rehabilitation methods surveyed were not exhaustive and did not include other commonly utilized rehabilitation modalities such as acupuncture, chiropractic treatment, massage, therapeutic ultrasound, underwater treadmill, saltwater spa therapy, swimming, therapeutic shoeing, and electrotherapies such as transcutaneous electrical nerve stimulation (TENS) or neural electrical muscle stimulation (NEMS). The case scenarios selected were meant to mimic common equine orthopedic surgical cases including different tissue types and elective versus emergency procedures; however, they are not likely to reflect management and rehabilitation modalities used for all equine orthopedic surgical cases. The septic joint case included intra-operative images of a septic tarsocrural joint but did not specifically state in the description that the case was a septic tarsocrural joint. The participants management strategies and responses may have differed based on the joint affected in the scenario and because it was not stated, this is a limitation. Additionally, only ACVS and ACVSMR diplomates were surveyed, and these results may not reflect opinions of European surgery or sports medicine and rehabilitation specialties or general practitioners that may be treating these cases. Most of the respondents were ACVS diplomates, therefore these results may not fully reflect the opinions of ACVSMR diplomates. Additionally, the inherent bias and low response rate that typically occur with online surveys must also be considered.

Despite the limitations associated with this study, results of this survey provide insight on postoperative management recommended by ACVS and ACVSMR diplomates for the equine orthopedic surgical cases provided. Some, but few, differences were identified in recommendations between ACVS and ACVSMR diplomates and are likely due to a variety of case and clinician factors. Differences were identified in management recommendations between the surgical case scenarios and likely reflect differences in tissue type affected by the surgical procedures, differences in healing of these tissues, and differing goals of the surgical procedures. The information presented here serves as a baseline for common postoperative management methods utilized and highlights areas for further investigation, education, and resident and diplomate training.

## Data Availability

The original contributions presented in the study are included in the article/Supplementary material, further inquiries can be directed to the corresponding author.
