# Supplementary material for: Postoperative management following equine orthopedic surgery: a survey of diplomates of the ACVS and ACVSMR
Source: Front Vet Sci. 2025 Dec 5;12:1708401. doi: 10.3389/fvets.2025.1708401 (PMC12716337; doi:10.3389/fvets.2025.1708401)
Supplement: Supplementary file 1 [file Table_1.DOCX]

**Survey questions**

1. Years in practice
   1. <5 years
   2. 5-10 years
   3. 11-20 years
   4. > 20 years
2. Type of practice
   1. Private- equine only
   2. Private- mixed
   3. Private- large animal
   4. Academic- equine only
   5. Academic- large animal
   6. Industry
   7. Other
3. Specialty college
   1. American College of Veterinary Surgeons (ACVS)
   2. American College of Veterinary Sports Medicine & Rehabilitation (ACVSMR)
   3. ACVS & ACVSMR
4. Commonly treated disciplines:
   1. Sport horse (dressage, hunter jumper, show jumper, eventing, polo)
   2. Western performance (reining, barrel racing, cutting, roping, working cow)
   3. Show horses (western pleasure, English pleasure, halter, driving, saddleseat)
   4. Racehorse
   5. Pleasure horse
   6. Combination of the above disciplines

**For the following questions, please consider management of a 2-3 year old with a simple osteochondral fragment (dorsal proximal P1 fragment, OCD of the tarsocrural joint) that is destined to be used as an athlete (racing, dressage, jumping, working western, show horse).**

1. When considering post operative management of cases undergoing simple arthroscopy for what % of cases are you prescribing an NSAID?
   1. 100%
   2. 75-99%
   3. 50-74%
   4. 25-49%
   5. 0-24%
2. When prescribing an NSAID for the post operative management of cases undergoing simple arthroscopy, what is your NSAID of choice?
   1. Phenylbutazone
   2. Flunixin meglumine
   3. Firocoxib
   4. Other?
   5. Prescribe phenylbutazone and flunixin meglumine with similar frequency
   6. Prescribe the above NSAIDs with similar frequency
3. When prescribing an NSAID for the post operative management of cases undergoing simple arthroscopy, what is the typical amount of time you prescribe the NSAID?
   1. 0-3 days
   2. 4-7 days
   3. 8-12 days
   4. 13-14 days
   5. 15-21 days
   6. > 21 days
4. When considering post operative management of cases undergoing simple arthroscopy, what is the typical amount of time you prescribe strict stall rest?
   1. 0-2 weeks
   2. 3-4 weeks
   3. 5-6 weeks
   4. 7-8 weeks
   5. 9-10 weeks
   6. 11-12 weeks
   7. 13-15 weeks
   8. >16 weeks
5. When considering post operative management of cases undergoing simple arthroscopy, do you recommend handwalking exercise?
   1. Yes
   2. No
6. When considering post operative management of cases undergoing simple arthroscopy, when do you **INITIATE** handwalking exercise?
   1. 0-2 weeks PO
   2. 3-4 weeks PO
   3. 5-6 weeks PO
   4. 7-8 weeks PO
   5. 9-10 weeks PO
   6. 11-12 weeks PO
   7. 13-15 weeks PO
   8. >16 weeks PO
7. When considering post operative management of cases undergoing simple arthroscopy, what is the **TOTAL** amount of time you recommend handwalking exercise?
   1. 0-2 weeks
   2. 3-4 weeks
   3. 5-6 weeks
   4. 7-8 weeks
   5. 9-10 weeks
   6. 11-12 weeks
   7. 13-15 weeks
   8. >16 weeks
8. When considering post operative management of cases undergoing simple arthroscopy, do you recommend small paddock turnout exercise?
   1. Yes
   2. No
9. When considering post operative management of cases undergoing simple arthroscopy, when do you **INITIATE** small paddock turnout?
   1. 0-2 weeks PO
   2. 3-4 weeks
   3. 5-6 weeks
   4. 7-8 weeks
   5. 9-10 weeks
   6. 11-12 weeks
   7. 13-15 weeks
   8. >16 weeks
10. When considering post operative management of cases undergoing simple arthroscopy, what is the **TOTAL** amount of time you recommend small paddock turnout?
    1. 0-2 weeks
    2. 3-4 weeks
    3. 5-6 weeks
    4. 7-8 weeks
    5. 9-10 weeks
    6. 11-12 weeks
    7. 13-15 weeks
    8. >16 weeks
11. When considering post operative management of cases undergoing simple arthroscopy, when do you **INITIATE** return to full or normal turnout?
    1. 0-2 weeks PO
    2. 3-4 weeks
    3. 5-6 weeks
    4. 7-8 weeks
    5. 9-10 weeks
    6. 11-12 weeks
    7. 13-15 weeks
    8. >16 weeks
12. When considering post operative management of cases undergoing simple arthroscopy, when do you **INITIATE** return to ridden exercise and/or training?
    1. 0-2 weeks PO
    2. 3-4 weeks
    3. 5-6 weeks
    4. 7-8 weeks
    5. 9-10 weeks
    6. 11-12 weeks
    7. 13-15 weeks
    8. >16 weeks
13. When considering post operative management of cases undergoing simple arthroscopy do you recommend any intra-articular therapy?
    1. Yes
    2. No
14. When considering post operative management of cases undergoing simple arthroscopy, when do you recommend intra-articular therapy?
    1. 0-2 weeks PO
    2. 3-4 weeks
    3. 5-6 weeks
    4. 7-8 weeks
    5. 9-10 weeks
    6. 11-12 weeks
    7. 13-15 weeks
    8. >16 weeks
15. When considering post operative management of cases undergoing simple arthroscopy, what intra-articular therapy do you most commonly recommend?
    1. Hyaluronate sodium (Hyvisc, Hyalovet, etc)
    2. Polysulfated glycosaminoglycan (Adequan)
    3. Corticosteroids (betamethasone, triamcinolone, methylprednisolone)
    4. Autologous conditioned serum (Interleukin-1 receptor antagonist protein/IRAP)
    5. Autologous protein solution (ProStride)
    6. Platelet rich plasma (ACP, ProVet, Restigen, Rebound)
    7. Alpha 2 macroglobulin
    8. Stem cell therapy (Bone marrow derived, adipose derived)
    9. Polyacrylamide hydrogel (NoltrexVet, Arthramid)
    10. Other
16. When considering post operative management of cases undergoing simple arthroscopy, do you recommend bandaging?
    1. Yes
    2. No
17. When considering post operative management of cases undergoing simple arthroscopy, what is the **TOTAL** amount of time you recommend bandaging?
    1. 0-2 weeks
    2. 3-4 weeks
    3. 5-6 weeks
    4. 7-8 weeks
    5. 9-10 weeks
    6. 11-12 weeks
    7. 13-15 weeks
    8. >16 weeks
18. When considering post operative management of cases undergoing simple arthroscopy, do you recommend cryotherapy?
    1. Yes
    2. No
19. When considering post operative management of cases undergoing simple arthroscopy, what type of cryotherapy do you recommend?
    1. Ice water immersion
    2. Commercial dry sleeve cryotherapy/compression
    3. Commercial cryotherapy boot
20. When considering post operative management of cases undergoing simple arthroscopy, what is the **TOTAL** amount of time you recommend cryotherapy?
    1. 0-2 weeks
    2. 3-4 weeks
    3. 5-6 weeks
    4. 7-8 weeks
    5. 9-10 weeks
    6. 11-12 weeks
    7. 13-15 weeks
    8. >16 weeks
21. When considering post operative management of cases undergoing simple arthroscopy, do you recommend core stabilization/strengthening exercises?
    1. Yes
    2. No
22. When considering post operative management of cases undergoing simple arthroscopy, what is the **TOTAL** amount of time you recommend core stabilization/strengthening exercises?
    1. 0-2 weeks
    2. 3-4 weeks
    3. 5-6 weeks
    4. 7-8 weeks
    5. 9-10 weeks
    6. 11-12 weeks
    7. 13-15 weeks
    8. >16 weeks
23. When considering post operative management of cases undergoing simple arthroscopy, do you recommend laser therapy?
    1. Yes
    2. No
24. When considering post operative management of cases undergoing simple arthroscopy, what type of laser therapy do you recommend?
    1. Low level (Class 3)
    2. High level (Class 4)
25. When considering post operative management of cases undergoing simple arthroscopy, what is the **TOTAL** amount of time you recommend laser therapy?
    1. 0-2 weeks
    2. 3-4 weeks
    3. 5-6 weeks
    4. 7-8 weeks
    5. 9-10 weeks
    6. 11-12 weeks
    7. 13-15 weeks
    8. >16 weeks
26. When considering post operative management of cases undergoing simple arthroscopy, do you recommend pulsed electromagnetic field/PEMF therapy?
    1. Yes
    2. No
27. When considering post operative management of cases undergoing simple arthroscopy, what type of pulsed electromagnetic field/PEMF therapy do you recommend?
    1. Blanket
    2. Wand
    3. Other
28. When considering post operative management of cases undergoing simple arthroscopy, what is the **TOTAL** amount of time you recommend PEMF therapy?
    1. 0-2 weeks
    2. 3-4 weeks
    3. 5-6 weeks
    4. 7-8 weeks
    5. 9-10 weeks
    6. 11-12 weeks
    7. 13-15 weeks
    8. >16 weeks
29. When considering post operative management of cases undergoing simple arthroscopy, do you recommend shockwave therapy?
    1. Yes
    2. No
30. When considering post operative management of cases undergoing simple arthroscopy, what is the **TOTAL** amount of time you recommend shockwave therapy?
    1. 0-2 weeks
    2. 3-4 weeks
    3. 5-6 weeks
    4. 7-8 weeks
    5. 9-10 weeks
    6. 11-12 weeks
    7. 13-15 weeks
    8. >16 weeks
31. When considering post operative management of cases undergoing simple arthroscopy, do you recommend range of motion exercises (ie. passive flexion/extension; walking over ground poles)?
    1. Yes
    2. No
32. When considering post operative management of cases undergoing simple arthroscopy, what is the **TOTAL** amount of time you recommend range of motion exercises (ie. passive flexion/extension; walking over ground poles)?
    1. 0-2 weeks
    2. 3-4 weeks
    3. 5-6 weeks
    4. 7-8 weeks
    5. 9-10 weeks
    6. 11-12 weeks
    7. 13-15 weeks
    8. >16 weeks

**For the following questions, please consider management of a middle aged horse that has been diagnosed with an acute (<6 hours old) septic joint secondary to a puncture wound.**

1. When considering post operative management of cases undergoing arthroscopy for acute joint sepsis, for which % of cases are you prescribing an NSAID?
   1. 100%
   2. 75-99%
   3. 50-74%
   4. 25-49%
   5. 0-24%
2. When prescribing an NSAID for the post operative management of cases undergoing arthroscopy for acute joint sepsis, what is your NSAID of choice?
   1. Phenylbutazone
   2. Flunixin meglumine
   3. Firocoxib
   4. Other?
   5. Prescribe phenylbutazone and flunixin meglumine with similar frequency
   6. Prescribe the above NSAIDs with similar frequency
3. When prescribing an NSAID for the post operative management of cases undergoing arthroscopy for acute joint sepsis, what is the typical amount of time you prescribe the NSAID?
   1. 0-3 days
   2. 4-7 days
   3. 8-12 days
   4. 13-14 days
   5. 15-21 days
   6. > 21 days
4. When considering post operative management of cases undergoing arthroscopy for acute joint sepsis, what is the typical amount of time you prescribe strict stall rest?
   1. 0-2 weeks
   2. 3-4 weeks
   3. 5-6 weeks
   4. 7-8 weeks
   5. 9-10 weeks
   6. 11-12 weeks
   7. 13-15 weeks
   8. >16 weeks
5. When considering post operative management of cases undergoing arthroscopy for acute joint sepsis, do you recommend handwalking exercise?
   1. Yes
   2. No
6. When considering post operative management of cases undergoing arthroscopy for acute joint sepsis, when do you **INITIATE** handwalking exercise?
   1. 0-2 weeks PO
   2. 3-4 weeks PO
   3. 5-6 weeks PO
   4. 7-8 weeks PO
   5. 9-10 weeks PO
   6. 11-12 weeks PO
   7. 13-15 weeks PO
   8. >16 weeks PO
7. When considering post operative management of cases undergoing arthroscopy for acute joint sepsis, what is the **TOTAL** amount of time you recommend handwalking exercise?
   1. 0-2 weeks
   2. 3-4 weeks
   3. 5-6 weeks
   4. 7-8 weeks
   5. 9-10 weeks
   6. 11-12 weeks
   7. 13-15 weeks
   8. >16 weeks
8. When considering post operative management of cases undergoing arthroscopy for acute joint sepsis, do you recommend small paddock turnout exercise?
   1. Yes
   2. No
9. When considering post operative management of cases undergoing arthroscopy for acute joint sepsis, when do you **INITIATE** small paddock turnout?
   1. 0-2 weeks PO
   2. 3-4 weeks
   3. 5-6 weeks
   4. 7-8 weeks
   5. 9-10 weeks
   6. 11-12 weeks
   7. 13-15 weeks
   8. >16 weeks
10. When considering post operative management of cases undergoing arthroscopy for acute joint sepsis, what is the **TOTAL** amount of time you recommend small paddock turnout?
    1. 0-2 weeks
    2. 3-4 weeks
    3. 5-6 weeks
    4. 7-8 weeks
    5. 9-10 weeks
    6. 11-12 weeks
    7. 13-15 weeks
    8. >16 weeks
11. When considering post operative management of cases undergoing arthroscopy for acute joint sepsis, when do you **INITIATE** return to full or normal turnout?
    1. 0-2 weeks PO
    2. 3-4 weeks
    3. 5-6 weeks
    4. 7-8 weeks
    5. 9-10 weeks
    6. 11-12 weeks
    7. 13-15 weeks
    8. >16 weeks
12. When considering post operative management of cases undergoing arthroscopy for acute joint sepsis, when do you **INITIATE** return to ridden exercise and/or training?
    1. 0-2 weeks PO
    2. 3-4 weeks
    3. 5-6 weeks
    4. 7-8 weeks
    5. 9-10 weeks
    6. 11-12 weeks
    7. 13-15 weeks
    8. >16 weeks
13. When considering post operative management of cases undergoing arthroscopy for acute joint sepsis, do you recommend any intra-articular therapy?
    1. Yes
    2. No
14. When considering post operative management of cases undergoing arthroscopy for acute joint sepsis, when do you recommend intra-articular therapy?
    1. 0-2 weeks PO
    2. 3-4 weeks
    3. 5-6 weeks
    4. 7-8 weeks
    5. 9-10 weeks
    6. 11-12 weeks
    7. 13-15 weeks
    8. >16 weeks
15. When considering post operative management of cases undergoing arthroscopy for acute joint sepsis, what intra-articular therapy do you most commonly recommend?
    1. Hyaluronate sodium (Hyvisc, Hyalovet, etc)
    2. Polysulfated glycosaminoglycan (Adequan)
    3. Corticosteroids (betamethasone, triamcinolone, methylprednisolone)
    4. Autologous conditioned serum (Interleukin-1 receptor antagonist protein/IRAP)
    5. Autologous protein solution (ProStride)
    6. Platelet rich plasma (ACP, ProVet, Restigen, Rebound)
    7. Alpha 2 macroglobulin
    8. Stem cell therapy (Bone marrow derived, adipose derived)
    9. Polyacrylamide hydrogel (NoltrexVet, Arthramid)
    10. Other
16. When considering post operative management of cases undergoing arthroscopy for acute joint sepsis, do you recommend bandaging?
    1. Yes
    2. No
17. When considering post operative management of cases undergoing arthroscopy for acute joint sepsis, what is the **TOTAL** amount of time you recommend bandaging?
    1. 0-2 weeks
    2. 3-4 weeks
    3. 5-6 weeks
    4. 7-8 weeks
    5. 9-10 weeks
    6. 11-12 weeks
    7. 13-15 weeks
    8. >16 weeks
18. When considering post operative management of cases undergoing arthroscopy for acute joint sepsis, do you recommend cryotherapy?
    1. Yes
    2. No
19. When considering post operative management of cases undergoing arthroscopy for acute joint sepsis, what type of cryotherapy do you recommend?
    1. Ice water immersion
    2. Commercial dry sleeve cryotherapy/compression
    3. Commercial cryotherapy boot
    4. Other
20. When considering post operative management of cases undergoing arthroscopy for acute joint sepsis, what is the **TOTAL** amount of time you recommend cryotherapy?
    1. 0-2 weeks
    2. 3-4 weeks
    3. 5-6 weeks
    4. 7-8 weeks
    5. 9-10 weeks
    6. 11-12 weeks
    7. 13-15 weeks
    8. >16 weeks
21. When considering post operative management of cases undergoing arthroscopy for acute joint sepsis, do you recommend core stabilization/strengthening exercises?
    1. Yes
    2. No
22. When considering post operative management of cases undergoing arthroscopy for acute joint sepsis, what is the **TOTAL** amount of time you recommend core stabilization/strengthening exercises?
    1. 0-2 weeks
    2. 3-4 weeks
    3. 5-6 weeks
    4. 7-8 weeks
    5. 9-10 weeks
    6. 11-12 weeks
    7. 13-15 weeks
    8. >16 weeks
23. When considering post operative management of cases undergoing arthroscopy for acute joint sepsis, do you recommend laser therapy?
    1. Yes
    2. No
24. When considering post operative management of cases undergoing arthroscopy for acute joint sepsis, what type of laser therapy do you recommend?
    1. Low level (Class 3)
    2. High level (Class 4)
25. When considering post operative management of cases undergoing arthroscopy for acute joint sepsis, what is the **TOTAL** amount of time you recommend laser therapy?
    1. 0-2 weeks
    2. 3-4 weeks
    3. 5-6 weeks
    4. 7-8 weeks
    5. 9-10 weeks
    6. 11-12 weeks
    7. 13-15 weeks
    8. >16 weeks
26. When considering post operative management of cases undergoing arthroscopy for acute joint sepsis, do you recommend pulsed electromagnetic field/PEMF therapy?
    1. Yes
    2. No
27. When considering post operative management of cases undergoing arthroscopy for acute joint sepsis, what type of pulsed electromagnetic field/PEMF therapy do you recommend?
    1. Blanket
    2. Wand
    3. Other
28. When considering post operative management of cases undergoing arthroscopy for acute joint sepsis, what is the **TOTAL** amount of time you recommend PEMF therapy?
    1. 0-2 weeks
    2. 3-4 weeks
    3. 5-6 weeks
    4. 7-8 weeks
    5. 9-10 weeks
    6. 11-12 weeks
    7. 13-15 weeks
    8. >16 weeks
29. When considering post operative management of cases undergoing arthroscopy for acute joint sepsis, do you recommend shockwave therapy?
    1. Yes
    2. No
30. When considering post operative management of cases undergoing arthroscopy for acute joint sepsis, what is the **TOTAL** amount of time you recommend shockwave therapy?
    1. 0-2 weeks
    2. 3-4 weeks
    3. 5-6 weeks
    4. 7-8 weeks
    5. 9-10 weeks
    6. 11-12 weeks
    7. 13-15 weeks
    8. >16 weeks
31. When considering post operative management of cases undergoing arthroscopy for acute joint sepsis, do you recommend range of motion exercises (ie. passive flexion/extension; walking over ground poles)?
    1. Yes
    2. No
32. When considering post operative management of cases undergoing arthroscopy for acute joint sepsis, what is the **TOTAL** amount of time you recommend range of motion exercises (ie. passive flexion/extension; walking over ground poles)?
    1. 0-2 weeks
    2. 3-4 weeks
    3. 5-6 weeks
    4. 7-8 weeks
    5. 9-10 weeks
    6. 11-12 weeks
    7. 13-15 weeks
    8. >16 weeks

**For the following questions, please consider management of a middle aged horse undergoing a simple tenoscopic procedure of the digital flexor tendon sheath (DFTS) such as a mild tear of the DDFT and/or annular ligament desmotomy. The owners would like to return the horse its previous level of athletic function (racing, dressage, jumping, working western, show horse).**

1. When considering post operative management of a case undergoing simple DFTS tenoscopy, for which % of cases are you prescribing an NSAID?
   1. 100%
   2. 75-99%
   3. 50-74%
   4. 25-49%
   5. 0-24%
2. When prescribing an NSAID for the post operative management of a case undergoing simple DFTS tenoscopy, what is your NSAID of choice?
   1. Phenylbutazone
   2. Flunixin meglumine
   3. Firocoxib
   4. Other?
   5. Prescribe phenylbutazone and flunixin meglumine with similar frequency
   6. Prescribe the above NSAIDs with similar frequency
3. When prescribing an NSAID for the post operative management of a case undergoing simple DFTS tenoscopy, what is the typical amount of time you prescribe the NSAID?
   1. 0-3 days
   2. 4-7 days
   3. 8-12 days
   4. 13-14 days
   5. 15-21 days
   6. > 21 days
4. When considering post operative management of a case undergoing simple DFTS tenoscopy, what is the typical amount of time you prescribe strict stall rest?
   1. 0-2 weeks
   2. 3-4 weeks
   3. 5-6 weeks
   4. 7-8 weeks
   5. 9-10 weeks
   6. 11-12 weeks
   7. 13-15 weeks
   8. >16 weeks
5. When considering post operative management of a case undergoing simple DFTS tenoscopy, do you recommend handwalking exercise?
   1. Yes
   2. No
6. When considering post operative management of a case undergoing simple DFTS tenoscopy, when do you **INITIATE** handwalking exercise?
   1. 0-2 weeks PO
   2. 3-4 weeks PO
   3. 5-6 weeks PO
   4. 7-8 weeks PO
   5. 9-10 weeks PO
   6. 11-12 weeks PO
   7. 13-15 weeks PO
   8. >16 weeks PO
7. When considering post operative management of a case undergoing simple DFTS tenoscopy, what is the **TOTAL** amount of time you recommend handwalking exercise?
   1. 0-2 weeks
   2. 3-4 weeks
   3. 5-6 weeks
   4. 7-8 weeks
   5. 9-10 weeks
   6. 11-12 weeks
   7. 13-15 weeks
   8. >16 weeks
8. When considering post operative management of a case undergoing simple DFTS tenoscopy, do you recommend small paddock turnout exercise?
   1. Yes
   2. No
9. When considering post operative management of a case undergoing simple DFTS tenoscopy, when do you **INITIATE** small paddock turnout?
   1. 0-2 weeks PO
   2. 3-4 weeks
   3. 5-6 weeks
   4. 7-8 weeks
   5. 9-10 weeks
   6. 11-12 weeks
   7. 13-15 weeks
   8. >16 weeks
10. When considering post operative management of a case undergoing simple DFTS tenoscopy, what is the **TOTAL** amount of time you recommend small paddock turnout?
    1. 0-2 weeks
    2. 3-4 weeks
    3. 5-6 weeks
    4. 7-8 weeks
    5. 9-10 weeks
    6. 11-12 weeks
    7. 13-15 weeks
    8. >16 weeks
11. When considering post operative management of a case undergoing simple DFTS tenoscopy, when do you **INITIATE** return to full or normal turnout?
    1. 0-2 weeks PO
    2. 3-4 weeks
    3. 5-6 weeks
    4. 7-8 weeks
    5. 9-10 weeks
    6. 11-12 weeks
    7. 13-15 weeks
    8. >16 weeks
12. When considering post operative management of a case undergoing simple DFTS tenoscopy, when do you **INITIATE** return to ridden exercise and/or training?
    1. 0-2 weeks PO
    2. 3-4 weeks
    3. 5-6 weeks
    4. 7-8 weeks
    5. 9-10 weeks
    6. 11-12 weeks
    7. 13-15 weeks
    8. >16 weeks
13. When considering post operative management of a case undergoing simple DFTS tenoscopy, do you recommend any intra-thecal therapy?
    1. Yes
    2. No
14. When considering post operative management of a case undergoing simple DFTS tenoscopy, when do you recommend intra-thecal therapy?
    1. 0-2 weeks PO
    2. 3-4 weeks
    3. 5-6 weeks
    4. 7-8 weeks
    5. 9-10 weeks
    6. 11-12 weeks
    7. 13-15 weeks
    8. >16 weeks
15. When considering post operative management of a case undergoing simple DFTS tenoscopy, what intra-thecal therapy do you most commonly recommend?
    1. Hyaluronate sodium (Hyvisc, Hyalovet, etc)
    2. Polysulfated glycosaminoglycan (Adequan)
    3. Corticosteroids (betamethasone, triamcinolone, methylprednisolone)
    4. Autologous conditioned serum (Interleukin-1 receptor antagonist protein/IRAP)
    5. Autologous protein solution (ProStride)
    6. Platelet rich plasma (ACP, ProVet, Restigen, Rebound)
    7. Alpha 2 macroglobulin
    8. Stem cell therapy (Bone marrow derived, adipose derived)
    9. Polyacrylamide hydrogel (NoltrexVet, Arthramid)
    10. Other
16. When considering post operative management of a case undergoing simple DFTS tenoscopy, do you recommend bandaging?
    1. Yes
    2. No
17. When considering post operative management of a case undergoing simple DFTS tenoscopy, what is the **TOTAL** amount of time you recommend bandaging?
    1. 0-2 weeks
    2. 3-4 weeks
    3. 5-6 weeks
    4. 7-8 weeks
    5. 9-10 weeks
    6. 11-12 weeks
    7. 13-15 weeks
    8. >16 weeks
18. When considering post operative management of a case undergoing simple DFTS tenoscopy, do you recommend cryotherapy?
    1. Yes
    2. No
19. When considering post operative management of a case undergoing simple DFTS tenoscopy, what type of cryotherapy do you recommend?
    1. Ice water immersion
    2. Commercial dry sleeve cryotherapy/compression
    3. Commercial cryotherapy boot
    4. Other
20. When considering post operative management of a case undergoing simple DFTS tenoscopy, what is the **TOTAL** amount of time you recommend cryotherapy?
    1. 0-2 weeks
    2. 3-4 weeks
    3. 5-6 weeks
    4. 7-8 weeks
    5. 9-10 weeks
    6. 11-12 weeks
    7. 13-15 weeks
    8. >16 weeks
21. When considering post operative management of a case undergoing simple DFTS tenoscopy, do you recommend core stabilization/strengthening exercises?
    1. Yes
    2. No
22. When considering post operative management of a case undergoing simple DFTS tenoscopy, what is the **TOTAL** amount of time you recommend core stabilization/strengthening exercises?
    1. 0-2 weeks
    2. 3-4 weeks
    3. 5-6 weeks
    4. 7-8 weeks
    5. 9-10 weeks
    6. 11-12 weeks
    7. 13-15 weeks
    8. >16 weeks
23. When considering post operative management of a case undergoing simple DFTS tenoscopy, do you recommend laser therapy?
    1. Yes
    2. No
24. When considering post operative management of a case undergoing simple DFTS tenoscopy, what type of laser therapy do you recommend?
25. Low level (Class 3)
26. High level (Class 4)
27. When considering post operative management of a case undergoing simple DFTS tenoscopy, what is the **TOTAL** amount of time you recommend laser therapy?
28. 0-2 weeks
29. 3-4 weeks
30. 5-6 weeks
31. 7-8 weeks
32. 9-10 weeks
33. 11-12 weeks
34. 13-15 weeks
35. >16 weeks
36. When considering post operative management of a case undergoing simple DFTS tenoscopy, do you recommend pulsed electromagnetic field/PEMF therapy?
    1. Yes
    2. No
37. When considering post operative management of a case undergoing simple DFTS tenoscopy, what type of pulsed electromagnetic field/PEMF therapy do you recommend?
    1. Blanket
    2. Wand
    3. Other
38. When considering post operative management of a case undergoing simple DFTS tenoscopy, what is the **TOTAL** amount of time you recommend PEMF therapy?
    1. 0-2 weeks
    2. 3-4 weeks
    3. 5-6 weeks
    4. 7-8 weeks
    5. 9-10 weeks
    6. 11-12 weeks
    7. 13-15 weeks
    8. >16 weeks
39. When considering post operative management of a case undergoing simple DFTS tenoscopy, do you recommend shockwave therapy?
    1. Yes
    2. No
40. When considering post operative management of a case undergoing simple DFTS tenoscopy, what is the **TOTAL** amount of time you recommend shockwave therapy?
    1. 0-2 weeks
    2. 3-4 weeks
    3. 5-6 weeks
    4. 7-8 weeks
    5. 9-10 weeks
    6. 11-12 weeks
    7. 13-15 weeks
    8. >16 weeks
41. When considering post operative management of a case undergoing simple DFTS tenoscopy, do you recommend range of motion exercises (ie. passive flexion/extension; walking over ground poles)?
    1. Yes
    2. No
42. When considering post operative management of a case undergoing simple DFTS tenoscopy, what is the **TOTAL** amount of time you recommend range of motion exercises (ie. passive flexion/extension; walking over ground poles)?
    1. 0-2 weeks
    2. 3-4 weeks
    3. 5-6 weeks
    4. 7-8 weeks
    5. 9-10 weeks
    6. 11-12 weeks
    7. 13-15 weeks
    8. >16 weeks

**For the following questions, please consider management of a middle aged horse undergoing a bilateral deep branch of the lateral plantar neurectomy and fasciotomy for bilateral proximal suspensory disease. The owner would like to return the horse its previous level of athletic function (racing, dressage, jumping, working western, show horse).**

1. When considering post operative management for a case undergoing a bilateral deep branch of the lateral plantar neurectomy and fasciotomy, for which % of cases are you prescribing an NSAID?
   1. 100%
   2. 75-99%
   3. 50-74%
   4. 25-49%
   5. 0-24%
2. When prescribing an NSAID for a case undergoing a bilateral deep branch of the lateral plantar neurectomy and fasciotomy, what is your NSAID of choice?
   1. Phenylbutazone
   2. Flunixin meglumine
   3. Firocoxib
   4. Other?
   5. Prescribe phenylbutazone and flunixin meglumine with similar frequency
   6. Prescribe the above NSAIDs with similar frequency
3. When prescribing an NSAID for a case undergoing a bilateral deep branch of the lateral plantar neurectomy and fasciotomy, what is the typical amount of time you prescribe the NSAID?
   1. 0-3 days
   2. 4-7 days
   3. 8-12 days
   4. 13-14 days
   5. 15-21 days
   6. > 21 days
4. When considering post operative management for a case undergoing a bilateral deep branch of the lateral plantar neurectomy and fasciotomy, what is the typical amount of time you prescribe strict stall rest?
   1. 0-2 weeks
   2. 3-4 weeks
   3. 5-6 weeks
   4. 7-8 weeks
   5. 9-10 weeks
   6. 11-12 weeks
   7. 13-15 weeks
   8. >16 weeks
5. When considering post operative management for a case undergoing a bilateral deep branch of the lateral plantar neurectomy and fasciotomy, do you recommend handwalking exercise?
   1. Yes
   2. No
6. When considering post operative management for a case undergoing a bilateral deep branch of the lateral plantar neurectomy and fasciotomy, when do you **INITIATE** handwalking exercise?
   1. 0-2 weeks PO
   2. 3-4 weeks PO
   3. 5-6 weeks PO
   4. 7-8 weeks PO
   5. 9-10 weeks PO
   6. 11-12 weeks PO
   7. 13-15 weeks PO
   8. >16 weeks PO
7. When considering post operative management for a case undergoing a bilateral deep branch of the lateral plantar neurectomy and fasciotomy, what is the **TOTAL** amount of time you recommend handwalking exercise?
   1. 0-2 weeks
   2. 3-4 weeks
   3. 5-6 weeks
   4. 7-8 weeks
   5. 9-10 weeks
   6. 11-12 weeks
   7. 13-15 weeks
   8. >16 weeks
8. When considering post operative management for a case undergoing a bilateral deep branch of the lateral plantar neurectomy and fasciotomy, do you recommend small paddock turnout exercise?
   1. Yes
   2. No
9. When considering post operative management for a case undergoing a bilateral deep branch of the lateral plantar neurectomy and fasciotomy, when do you **INITIATE** small paddock turnout?
   1. 0-2 weeks PO
   2. 3-4 weeks
   3. 5-6 weeks
   4. 7-8 weeks
   5. 9-10 weeks
   6. 11-12 weeks
   7. 13-15 weeks
   8. >16 weeks
10. When considering post operative management for a case undergoing a bilateral deep branch of the lateral plantar neurectomy and fasciotomy, what is the **TOTAL** amount of time you recommend small paddock turnout?
    1. 0-2 weeks
    2. 3-4 weeks
    3. 5-6 weeks
    4. 7-8 weeks
    5. 9-10 weeks
    6. 11-12 weeks
    7. 13-15 weeks
    8. >16 weeks
11. When considering post operative management for a case undergoing a bilateral deep branch of the lateral plantar neurectomy and fasciotomy, when do you **INITIATE** return to full or normal turnout?
    1. 0-2 weeks PO
    2. 3-4 weeks
    3. 5-6 weeks
    4. 7-8 weeks
    5. 9-10 weeks
    6. 11-12 weeks
    7. 13-15 weeks
    8. >16 weeks
12. When considering post operative management for a case undergoing a bilateral deep branch of the lateral plantar neurectomy and fasciotomy, when do you **INITIATE** return to ridden exercise and/or training?
    1. 0-2 weeks PO
    2. 3-4 weeks
    3. 5-6 weeks
    4. 7-8 weeks
    5. 9-10 weeks
    6. 11-12 weeks
    7. 13-15 weeks
    8. >16 weeks
13. When considering post operative management for a case undergoing a bilateral deep branch of the lateral plantar neurectomy and fasciotomy, do you recommend any regenerative/biologic/alternative therapies?
    1. Yes
    2. No
14. When considering post operative management for a case undergoing a bilateral deep branch of the lateral plantar neurectomy and fasciotomy, when do you recommend regenerative/biologic/alternative therapies?
    1. 0-2 weeks PO
    2. 3-4 weeks
    3. 5-6 weeks
    4. 7-8 weeks
    5. 9-10 weeks
    6. 11-12 weeks
    7. 13-15 weeks
    8. >16 weeks
15. When considering post operative management for a case undergoing a bilateral deep branch of the lateral plantar neurectomy and fasciotomy, what regenerative/biologic/alternative therapy do you most commonly recommend?
    1. Hyaluronate sodium (Hyvisc, Hyalovet, etc)
    2. Polysulfated glycosaminoglycan (Adequan)
    3. Corticosteroids (betamethasone, triamcinolone, methylprednisolone)
    4. Autologous conditioned serum (Interleukin-1 receptor antagonist protein/IRAP)
    5. Autologous protein solution (ProStride)
    6. Platelet rich plasma (ACP, ProVet, Restigen, Rebound)
    7. Alpha 2 macroglobulin
    8. Stem cell therapy (Bone marrow derived, adipose derived)
    9. Polyacrylamide hydrogel (NoltrexVet, Arthramid)
    10. Other
16. When considering post operative management for a case undergoing a bilateral deep branch of the lateral plantar neurectomy and fasciotomy, do you recommend bandaging?
    1. Yes
    2. No
17. When considering post operative management for a case undergoing a bilateral deep branch of the lateral plantar neurectomy and fasciotomy, what is the **TOTAL** amount of time you recommend bandaging?
18. 0-2 weeks
19. 3-4 weeks
20. 5-6 weeks
21. 7-8 weeks
22. 9-10 weeks
23. 11-12 weeks
24. 13-15 weeks
25. >16 weeks
26. When considering post operative management for a case undergoing a bilateral deep branch of the lateral plantar neurectomy and fasciotomy, do you recommend cryotherapy?
    1. Yes
    2. No
27. When considering post operative management for a case undergoing a bilateral deep branch of the lateral plantar neurectomy and fasciotomy, what type of cryotherapy do you recommend?
    1. Ice water immersion
    2. Commercial dry sleeve cryotherapy/compression
    3. Commercial cryotherapy boot
    4. Other
28. When considering post operative management for a case undergoing a bilateral deep branch of the lateral plantar neurectomy and fasciotomy, what is the **TOTAL** amount of time you recommend cryotherapy?
    1. 0-2 weeks
    2. 3-4 weeks
    3. 5-6 weeks
    4. 7-8 weeks
    5. 9-10 weeks
    6. 11-12 weeks
    7. 13-15 weeks
    8. >16 weeks
29. When considering post operative management for a case undergoing a bilateral deep branch of the lateral plantar neurectomy and fasciotomy, do you recommend core stabilization/strengthening exercises?
30. Yes
31. No
32. When considering post operative management for a case undergoing a bilateral deep branch of the lateral plantar neurectomy and fasciotomy, what is the **TOTAL** amount of time you recommend core stabilization/strengthening exercises?
    1. 0-2 weeks
    2. 3-4 weeks
    3. 5-6 weeks
    4. 7-8 weeks
    5. 9-10 weeks
    6. 11-12 weeks
    7. 13-15 weeks
    8. >16 weeks
33. When considering post operative management for a case undergoing a bilateral deep branch of the lateral plantar neurectomy and fasciotomy, do you recommend laser therapy?
    1. Yes
    2. No
34. When considering post operative management for a case undergoing a bilateral deep branch of the lateral plantar neurectomy and fasciotomy, what type of laser therapy do you recommend?
35. Low level (Class 3)
36. High level (Class 4)
37. When considering post operative management for a case undergoing a bilateral deep branch of the lateral plantar neurectomy and fasciotomy, what is the **TOTAL** amount of time you recommend laser therapy?
38. 0-2 weeks
39. 3-4 weeks
40. 5-6 weeks
41. 7-8 weeks
42. 9-10 weeks
43. 11-12 weeks
44. 13-15 weeks
45. >16 weeks
46. When considering post operative management for a case undergoing a bilateral deep branch of the lateral plantar neurectomy and fasciotomy, do you recommend pulsed electromagnetic field/PEMF therapy?
    1. Yes
    2. No
47. When considering post operative management for a case undergoing a bilateral deep branch of the lateral plantar neurectomy and fasciotomy, what type of pulsed electromagnetic field/PEMF therapy do you recommend?
    1. Blanket
    2. Wand
    3. Other
48. When considering post operative management for a case undergoing a bilateral deep branch of the lateral plantar neurectomy and fasciotomy, what is the **TOTAL** amount of time you recommend PEMF therapy?
    1. 0-2 weeks
    2. 3-4 weeks
    3. 5-6 weeks
    4. 7-8 weeks
    5. 9-10 weeks
    6. 11-12 weeks
    7. 13-15 weeks
    8. >16 weeks
49. When considering post operative management for a case undergoing a bilateral deep branch of the lateral plantar neurectomy and fasciotomy, do you recommend shockwave therapy?
    1. Yes
    2. No
50. When considering post operative management for a case undergoing a bilateral deep branch of the lateral plantar neurectomy and fasciotomy, what is the **TOTAL** amount of time you recommend shockwave therapy?
    1. 0-2 weeks
    2. 3-4 weeks
    3. 5-6 weeks
    4. 7-8 weeks
    5. 9-10 weeks
    6. 11-12 weeks
    7. 13-15 weeks
    8. >16 weeks
51. When considering post operative management for a case undergoing a bilateral deep branch of the lateral plantar neurectomy and fasciotomy, do you recommend range of motion exercises (ie. passive flexion/extension; walking over ground poles)?
    1. Yes
    2. No
52. When considering post operative management for a case undergoing a bilateral deep branch of the lateral plantar neurectomy and fasciotomy, what is the **TOTAL** amount of time you recommend range of motion exercises (ie. passive flexion/extension; walking over ground poles)?
    1. 0-2 weeks
    2. 3-4 weeks
    3. 5-6 weeks
    4. 7-8 weeks
    5. 9-10 weeks
    6. 11-12 weeks
    7. 13-15 weeks
    8. >16 weeks
