# Supplementary material for: Postoperative management following equine orthopedic surgery: a survey of diplomates of the ACVS and ACVSMR
Source: Front Vet Sci. 2025 Dec 5;12:1708401. doi: 10.3389/fvets.2025.1708401 (PMC12716337; doi:10.3389/fvets.2025.1708401)
Supplement: Supplementary file 2 [file Presentation_1.PPTX]

## Slide 1
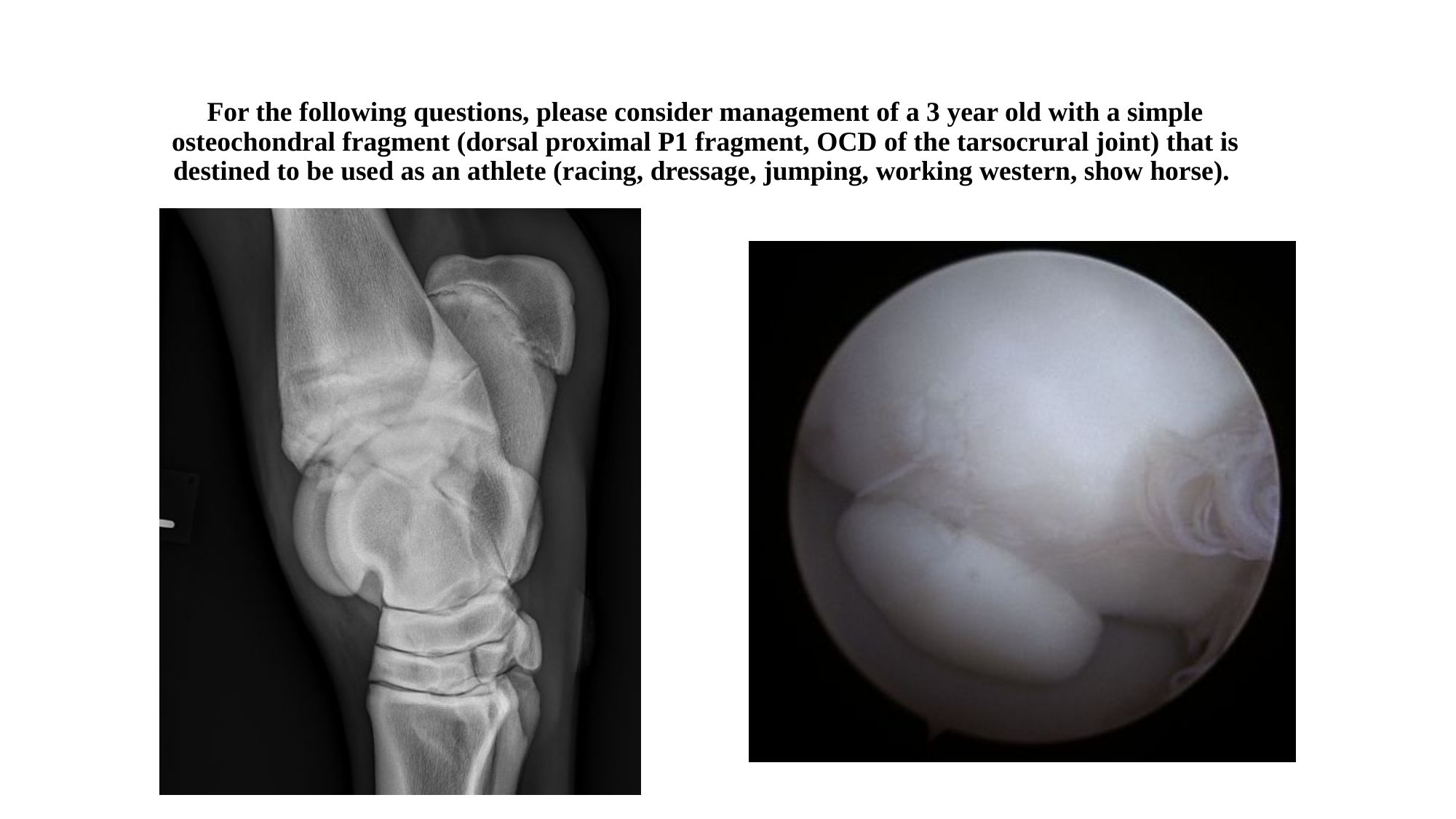

For the following questions, please consider management of a 3 year old with a simple osteochondral fragment (dorsal proximal P1 fragment, OCD of the tarsocrural joint) that is destined to be used as an athlete (racing, dressage, jumping, working western, show horse).

## Slide 2
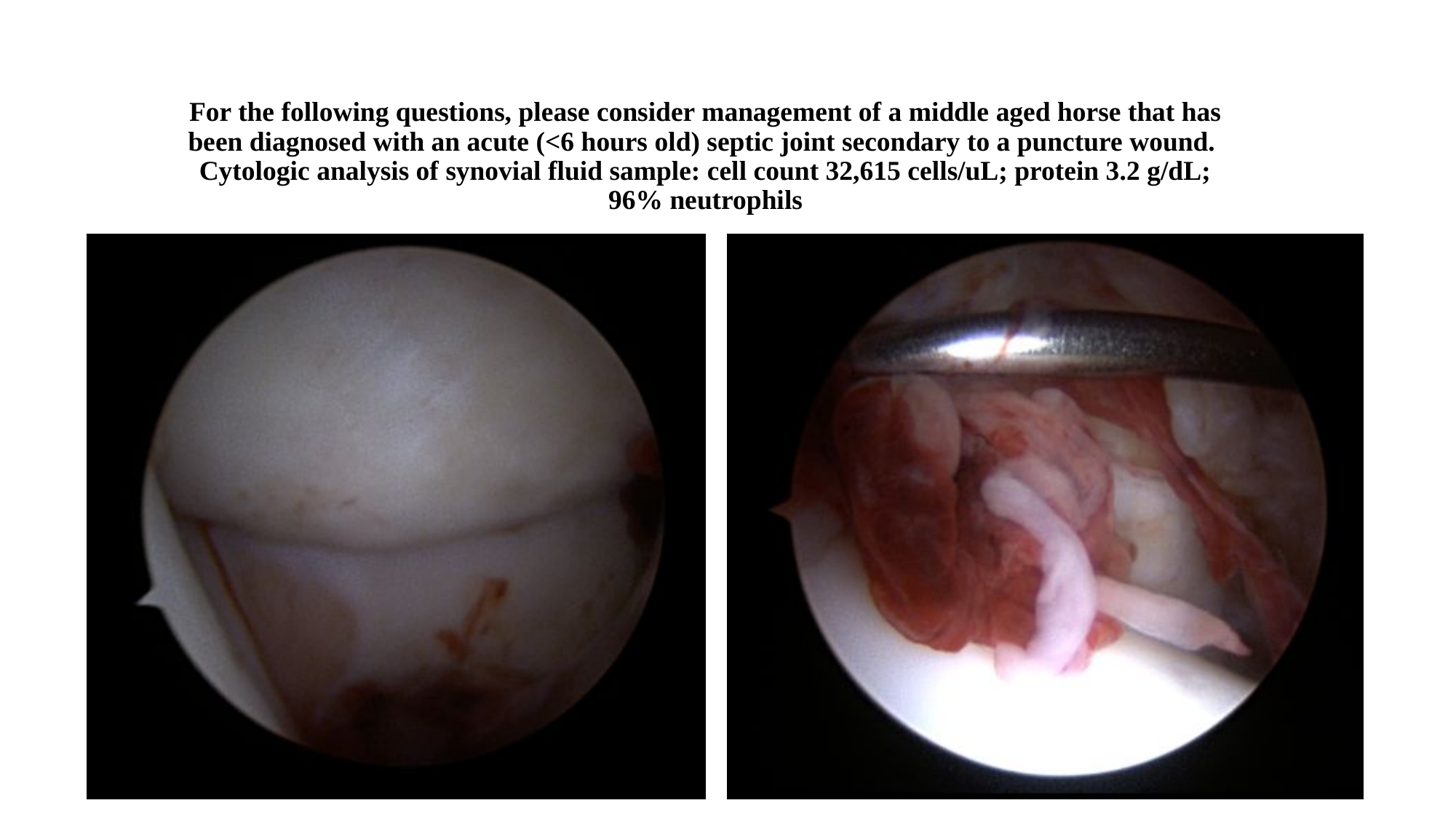

For the following questions, please consider management of a middle aged horse that has been diagnosed with an acute (<6 hours old) septic joint secondary to a puncture wound. Cytologic analysis of synovial fluid sample: cell count 32,615 cells/uL; protein 3.2 g/dL; 96% neutrophils

## Slide 3
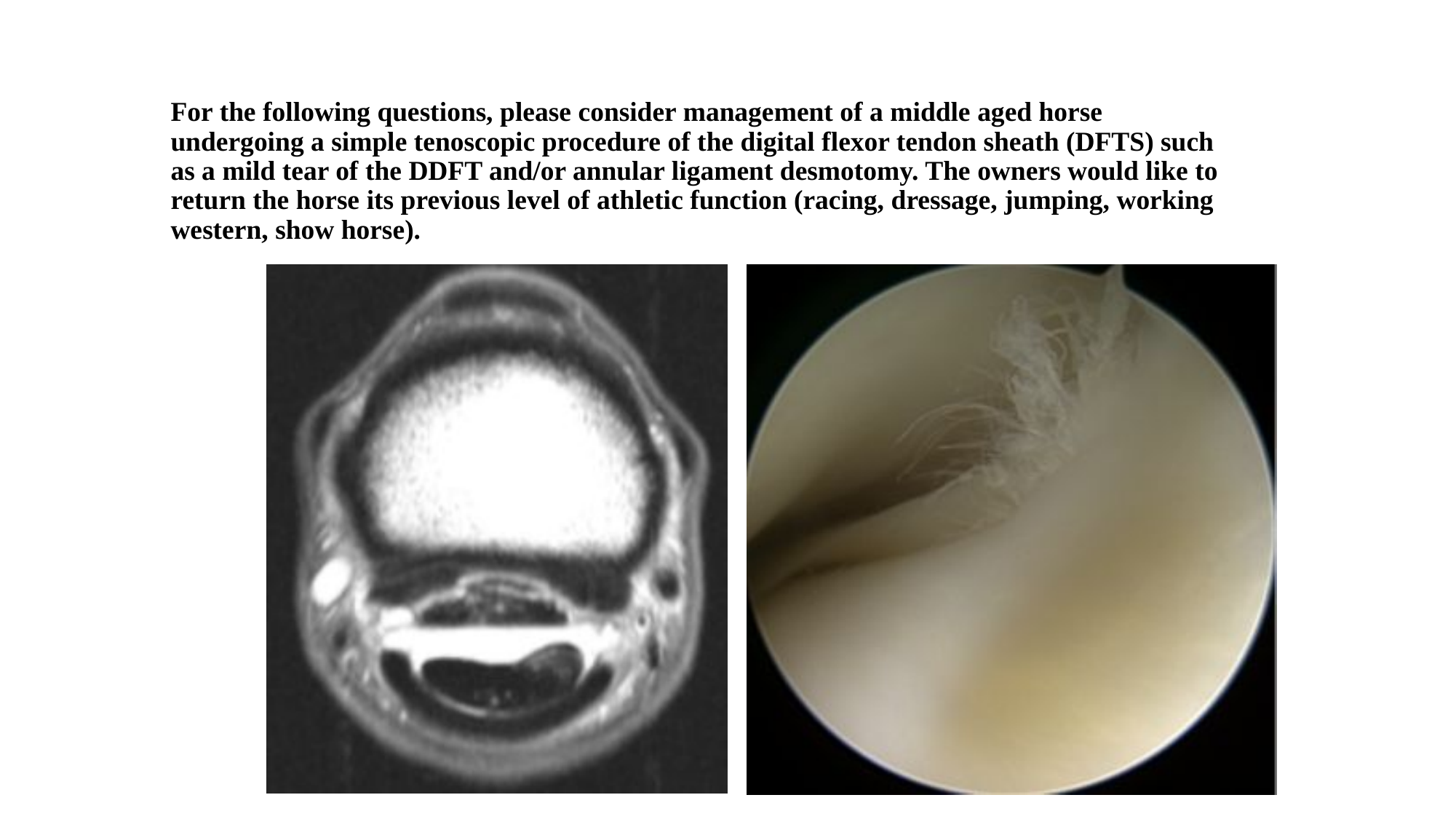

For the following questions, please consider management of a middle aged horse undergoing a simple tenoscopic procedure of the digital flexor tendon sheath (DFTS) such as a mild tear of the DDFT and/or annular ligament desmotomy. The owners would like to return the horse its previous level of athletic function (racing, dressage, jumping, working western, show horse).

## Slide 4
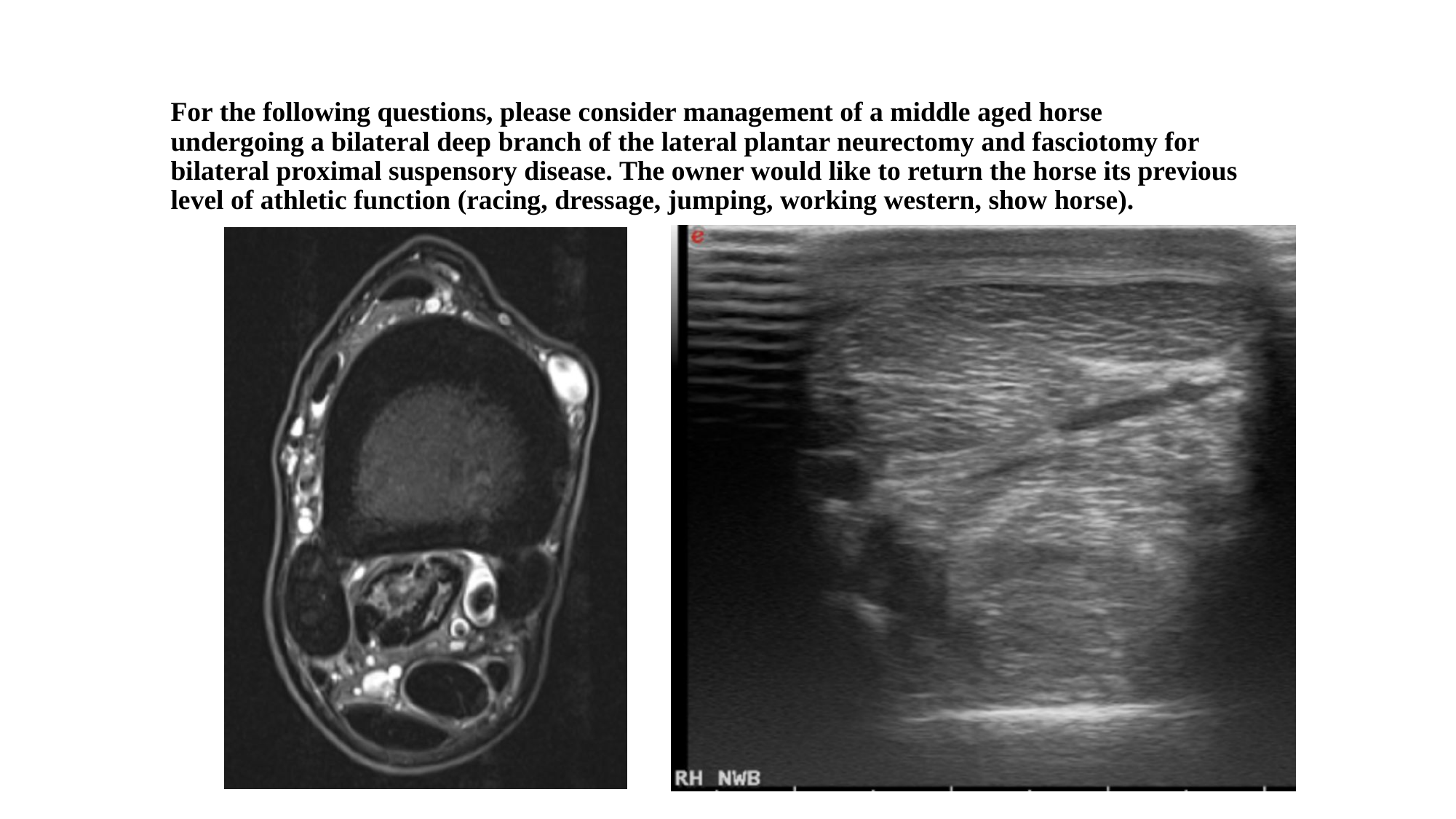

For the following questions, please consider management of a middle aged horse undergoing a bilateral deep branch of the lateral plantar neurectomy and fasciotomy for bilateral proximal suspensory disease. The owner would like to return the horse its previous level of athletic function (racing, dressage, jumping, working western, show horse).
